# Exergames improve cognitive function in older adults and their possible mechanisms: A systematic review

**DOI:** 10.7189/jogh.13.04177

**Published:** 2023-12-01

**Authors:** Yi Yang, Kun Wang, Shiqi Liu, Hengxu Liu, Tingran Zhang, Jiong Luo

**Affiliations:** Sports Drug Rehabilitation Research Center, School of Physical Education, Southwest University, Chongqing, China

## Abstract

**Objective:**

The degree of aging in China is deepening, leading to cognitive decline and seriously affecting the health status of the elderly. This article explores the benefits of exergames in improving cognitive function in older adults through a literature review, with aim of contributing to the development of healthy aging.

**Methods:**

Using PubMed, Web of Science, CNKI, dimensional spectrum, search for research papers from 2005 to April 2023 by using keywords such as a somatosensory game, cognitive function, execute function, working memory, aged and suppression control. PEDro Scale was used to check the quality of the literature.

**Results:**

A total of 16 papers were included in the review. Exergames improve the executive function of the elderly and support the relationship between exergames and cognitive performance in older adults. From a mechanistic perspective, somatosensory play promotes signal transduction of brain neurotrophin, thereby protecting the structure and function of neurons in specific brain regions and delays the decline of executive function in older adults as much as possible.

**Conclusions:**

It is recommended to use exergames as an intervention measure for the physical and mental health preservation of older adults. Suggest adopting personalised exergames in the future to explore the impact of cognitive and physical functions in elderly people, as well as measurable changes in brain structure.

Chinese experts' predictions on the current situation of dementia in China show that the incidence of elderly dementia will continue to rise from 2020 to 2049 and the prevalence rate of the entire population is 9.651% [1]. With the increasing trend of the elderly population in China, the health status of these elderly individuals is an essential issue of social and national concern. With the advancement of medical services, science and technology, a healthy aging society has achieved rapid development. However, the neurological diseases brought about by aging are urgent challenges that need to be addressed in the aging phenomenon of the elderly. Aging leads to a decline in cognitive function, which in turn leads to mild cognitive impairment and even dementia, seriously affecting the daily behaviour and health status of the elderly. Dementia is a neurodegenerative disease caused by aging. It will reduce the memory, decision-making and language expression abilities of the elderly [2].

There is no complete cure for dementia yet, but it has been confirmed that cognitive abilities in older adults improve through physical exercise intervention [3]. Previous studies by Wang Hongli et al. [2] have shown that delaying cognitive decline is the key to preventing dementia and aerobic exercise is an effective means to achieve it [4]. In the post-pandemic era, the concept of a healthy life is constantly ingrained in people's hearts and a healthy body is an inherent capital for a happy life. In addition, the rapid development of science and technology has led to significant changes in people's healthy lifestyles. It is worth noting that internet technology has improved the scientific home exercise of the elderly and has the characteristics of timely feedback and interaction with society. Among them, as a pre-screen interactive entertainment product, exergames have been continuously developed and have evolved, and are no longer limited to sedentary finger manipulation games, but rather full-body interactive physical activity games [5]. Participating in exergames can effectively increase energy expenditure in older Adult and improve physical and cognitive functions, among others [6], effectively achieving the goal of promoting physical and mental health [7]. Therefore, exergames involve physical activity, energy consumption and the regulation of bodily functions, which will improve the healthy physical fitness level of the elderly and effectively assist in their healthy aging.

Exergeme is an interactive product that combines body movements with technology and gaming, where individuals interact with virtual environments through their actions, requiring input from body movements and cognitive tasks (such as considering continuous feedback and quick decision-making) to control the output of game terminals during operation [8]. Nowadays, there are many types of body feeling games, with Nintendo Wii from Japan, Sony PlayStation from Japan, Xbox Kinect from the United States and Idong from China being the main types of body feeling games used in the field of sports rehabilitation [5,9]. At the same time, exergames can be divided into upper limb type games based on the part of the body that moves (such as bowling in Nintendo Wii, which only relies on the upper body's dominant movements for power). Lower limb exergames (such as dance machines, where lower limbs dominate participation), whole body exergames (such as Xbox Kinect, comprehensive body exercise programmes such as dance, Tai Chi, etc.). Active participation in physical games involves the energy expenditure of the elderly, who can control the game through their physical movements, which is the key difference from traditional electronic games [10]. It is worth noting that older adults are unable to participate in high-intensity exercise due to physical degradation caused by aging [3]. herefore, the majority of exergames for the elderly are aerobic exercises, and the best exercise prescription is for multiple components such as low-strength, coordination and sensitivity. In the face of rehabilitation treatment for special older adults, specially designed plans need to be adopted for treatment.

The intervention of exergames on the physical and mental health of older adults is a topic worth paying attention to both domestically and internationally. Exergames include cognitive motor dual task training and dual task interactive stimulation. This is a new strategy to improve the cognitive ability of elderly people [8]. Exergames have a positive impact on cognitive function and recovery of limb function in elderly people [11]. Previous studies have pointed out that combining physical exercise with cognitive stimulation may be a more effective strategy for improving cognitive skills in older adults compared to single-exercise training [12]. Therefore, exergames provide multiple sensory stimuli to older adults in a rich and complex gaming environment, providing multiple repetitions of goal-oriented tasks, thereby improving action learning and cognitive abilities. At the same time, exergames increase their physical activity and energy consumption, thereby improving the health and physical condition of the elderly [13,14]. In addition, by adjusting the different modes, levels and difficulty levels of the game, the interest and compliance of the elderly can be improved, coupled with the characteristics of convenient use, strong interactivity and low cost [15,16]. Furthermore, exergames can be used for exercise at home and for clinical applications, making them widely used in clinical treatment and scientific research [7].

In summary, exergames that utilise body-action interaction can effectively delay aging and reduce the risk of dementia. In addition, exergames also have positive effects on promoting the health of the elderly. Meanwhile, considering the increasing number of patients with cognitive impairment worldwide, it is necessary to better understand how physical exercise promotes cognition. This is also an important process in developing nondrug treatment strategies to improve the cognitive function of vulnerable groups. However, the effects of exergames vary greatly in different studies. In addition, the potential neurophysiological mechanisms of exergames on cognitive function in older adults are still unclear. Based on this, this article will further focus on exploring the benefits and mechanisms of exergames on cognitive function in older adults through a literature review, and propose relevant suggestions to assist the cause of healthy aging.

## METHODS

### Data sources

Using PubMed, Web of Science, China National Knowledge Infrastructure(CNKI) and Dimensional spectrum, we searched for research papers published in relevant domestic and international journals from 2005 to April 2021. We used the following keywords: somatosensory game, cognitive function, aged, execute function, working memory, suppression control.

### Selection criteria

We used four selection criteria: 1) the research subjects are composed of older adults aged 60 and above; 2) the experimental group has a strict exercise prescription design; 3) the exercise prescription for the experimental group must be based on exergames, while the control group can prescribe other exercises or not intervene; 4) the prescription design follows the standards of the American School of Sports Medicine (ACSM). The evaluation indexes mainly include cognitive ability, executive function, working memory, inhibition control.

### Literature exclusion criteria

We used three literature exclusion criteria: 1) non-English or non-Chinese literature; 2) repetitive and non-experimental studies; 3) literature unrelated to exercise prescriptions for exergames.

### Data intake quality assessment

1) The shortlisted literature is read in three stages. In the first stage, the researchers searched the database, browsed the titles and abstracts and conducted a preliminary screening of the retrieved literature. In the second stage, another researcher organised the literature and excluded duplicate literature. In the third stage, two researchers read all the literature to determine whether they met the inclusion criteria (if there was no consensus on any literature, it would be decided whether to include it after discussion).

2) Literature quality and empirical level. The PEDro scale was used to check each document and evaluate its research quality. The higher the score, the better the research quality of this document. Each document was scored independently by two researchers. If there are different scoring items, a consensus was reached after discussion. Due to the characteristics of the included papers, the therapists are required to provide treatment intervention in the research process. The highest total score may be nine for the items that cannot be single-blind for the therapists. Therefore, it is determined that those whose PEDro-scale score is greater than or equal to five are high-quality papers, and those whose score is less than or equal to four are low-quality papers.

**Figure 1:**
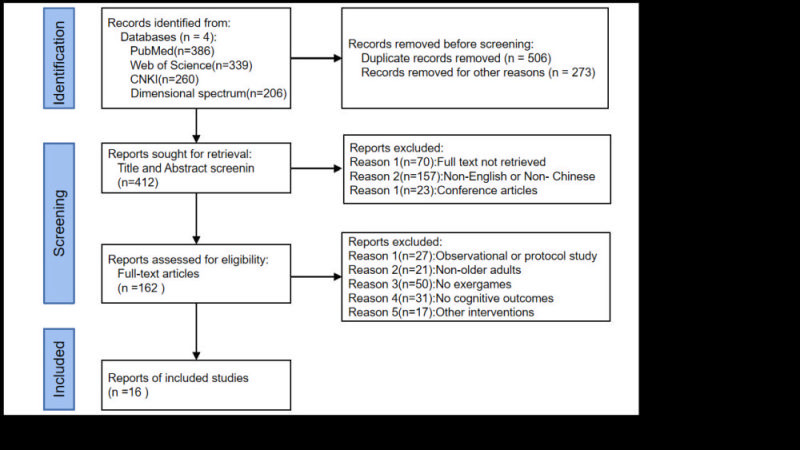
Flowchart regarding the selection process of the scientific studies.

The system search results are shown in **Figure 1**. A total of 1191 relevant articles were retrieved from four databases. We deleted 506 duplicate literature and selected 412 studies. According to the title and abstract, 162 full texts were obtained for further analysis, of which 146 were excluded because they did not meet the qualification criteria. Through the full-text analysis, 16 papers met the inclusion criteria and were included in this review.

## RESULTS

### Possible mechanism of exergames improving cognitive function in older adults

#### Somatosensory play promotes the release and expression of brain neurotrophin

More and more evidence suggests that physical activity provides an economical and efficient way to improve cognitive function in older adults, especially those prone to neurodegenerative diseases [4]. It is worth noting that brain-derived neurotrophin (BDNF), insulin-like growth factor 1 (IGF-1), vascular endothelial growth factor (VEGF) and other neurotrophins are considered key proteins mediating the downstream effects of exercise on brain and cognition [17]. This type of biomarker has been proven to increase in routine physical and cognitive activities and promote changes in brain structure by promoting the synaptic generation, neurogenesis and angiogenesis [18,19]. Therefore, under the combination of cognitive intervention and motor intervention, somatosensory play may change the signal of neurotrophin to maintain and enhance brain function, thereby changing the neuronal function and brain area structure of areas critical to cognition and playing a role in maintaining cognitive function under aging and neuropathological conditions.

##### Brain derived neurotrophin

Somatosensory play mediated brain-derived neurotrophin plays a positive role in the cognitive function of the elderly. Previous studies have shown that BDNF can cross the blood-brain barrier in a bidirectional manner and is a key molecule involved in a wide range of neurophysiological processes. Specifically, BDNF plays a key role in the growth and survival of neurons, that is, it promotes neuronal plasticity, which is critical to learning related synaptic plasticity and memory processes [20,21]. Research has shown that BDNF concentration is related to spatial memory performance and the volume of brain regions important for memory in healthy elderly individuals. A single exercise has been shown to increase the concentration of BDNF and it is intensity dependent and transient [22]. This affects memory by inducing changes in membrane receptor expression and translocation, as well as activating several pathways (PLC-y, PI3K, ERK). These pathways work together to promote cellular effects that affect synaptic plasticity [23]. In addition, exergames lasting for six weeks (twice a week/40 minutes) improved the “language” field of older adults and showed an improvement trend in the “abstract”, “memory and delayed recall” fields. The improvement of cognitive performance is associated with an increase in BDNF levels [21]. Elderly patients with mild cognitive impairment showed a significant increase in BDNF concentration and improvement in executive function after intervention in the “memory channel” interactive cycling somatosensory game [24]. There are also studies that suggest that long-term aerobic exercise can improve the basal concentration of BDNF levels or enhance the response of BDNF to exercise, thereby affecting its cognitive function, which is one of the effective pathways [4]. Therefore, long-term adherence to aerobic based somatosensory gaming types can certainly improve cognitive function in older Adult.

##### Insulin growth factor 1

Insulin like growth factor 1 (IGF-1) is an important nutrient factor in human growth and metabolic responses. Elevated levels of IGF-1 in the brain can improve neuronal proliferation, survival and plasticity. To protect nerves and promote angiogenesis. At the same time, insulin-like growth factor 1 (IGF-1) and BDNF cooperate to regulate the effect of exercise on neuroplasticity and mediate the expression of exercise-related BDNF mRNA in the brain [25]. They promote brain development, including cognitive function, by controlling factors related to synaptic plasticity [26]. Research has shown that during anaerobic exercise, resistance exercise stimulates the secretion of vascular endothelial growth factor and growth hormone by the pituitary gland and promotes the secretion of IGF-1 by the liver. It can regenerate the nerves in the hippocampus to improve the plasticity of synapses and memory function and has a positive effect on slowing down aging and improving memory function [27,28]. Empirical research also found that physical exercise can increase insulin growth factor levels in older adults [29]. Long term resistance exercise may have better effects [30]. In addition, exercise intervention can regulate the expression of IGF-1 in the brain and periphery, and then mediate the hippocampus to improve brain cognitive function [31]. In a study by Wall et al. [24], older adults participated in a three-month home trial of portable neural exergames (iPACES v2.0). Participants exercised by cycling and turning along a virtual memory lane. The results showed that the improvement of executive function in older adults was significantly correlated with the increase in IGF-1 expression. In a three-month family pilot clinical trial, it was found that exergames have an impact on patients with mild cognitive impairment after the intervention. Among them, IGF-1 mediates the improvement of delayed language memory and executive function in patients [32]. Therefore, improving cognitive function in older adults through exergames may be achieved by optimising the concentration and expression of IGF-1.

##### Vascular endothelial growth factor

Vascular endothelial growth factor (VEGF) is a signaling protein that stimulates angiogenesis. VEGF participates in the division and survival of endothelial cells, regulates angiogenesis, stimulates the generation of nerves and synapses, thus improving cognitive function [33-35]. Studies have shown that the characteristics of angiogenesis are changes in vascular function and structure, an increase in arterial diameter and an expansion of capillary networks, leading to an increase in blood flow [33]. Angiogenesis is precisely increased by VEGF in a complex process, partially controlled by IGF-1, as well as other energy-dependent processes regulated by available cellular oxygen and glucose [36]. However, similar to IGF-1, vascular endothelial growth factor also increases peripheral VEGF during exercise and some VEGF mediates exercise-induced angiogenesis and neurogenesis [37]. In addition, in animal experiments, exercise can inhibit the level of angiostatin mRNA and its expression, which is attributed to the enhanced VEGF signal during exercise [38]. Lactic acid is a key regulatory factor of VEGF; its activation can enhance cerebral vascular endothelial growth factor A (VEGFA) and cerebrovascular blood production. Animal experiments have also shown that VEGF released from exercise muscles mediates cerebral angiogenesis by activating lactate receptors, thereby increasing cerebral blood flow [35]. Therefore, VEGF improves cerebral blood flow in the structural adaptation of angiogenesis. In addition, VEGF can promote neurogenesis and synaptogenesis as an intermediate mechanism through the differentiation and proliferation of nerve cells. Kujach S et al. [19] also found that acute intermittent sprint exercise induces an increase in VEGF levels and VEGF expression levels play an important role in improving cognitive function [39]. Regular exercise training and acute exercise can increase the expression of VEGF and IGF-1. The increase in temporal lobe functional connectivity caused by exercise is related to changes in growth factors and may be enhanced by higher baseline VEGF [40].

In conclusion, somatosensory play may promote the level and expression of brain-derived neurotrophin, insulin-like growth factor and vascular endothelial growth factor. The three factors promote the differentiation and proliferation of neurons, induce cerebral angiogenesis, improve synaptic plasticity and enhance the resistance of the brain to functional and structural neurodegeneration, thus improving the cognitive function of the elderly. At the same time, the interaction of the three neurotrophins is also important. The emergence of BDNF increases the production of IGF-1 and IGF-1 also increases the binding affinity of BDNF with corresponding receptors. In addition, different forms of exercise (that is, different types of exergames) combined with different factors may promote changes in brain function through beneficial interactions of existing neurotrophin, hormones and other microbial parameters.

#### Exergames improve brain structure and function

In addition to improving the neurotrophin water that induces changes in brain structure, somatosensory play is also one of the ways to improve the cognitive function of the elderly. The brain structure is highly correlated with cognitive function. Physical exercise has also been shown to affect cognitive function by improving the structure of key brain regions main pathway is the atrophy of cognitive-related brain regions such as the hippocampus, lateral prefrontal cortex, caudate nucleus and cerebellar hemisphere caused by reverse aging [41,42].

##### Hippocampus

The hippocampus is located deep in the medial temporal lobe of the brain and is part of the limbic system. Its main functions include consolidating situational memory and situational dependent spatial learning, as well as regulating emotional behaviour [43]. Especially the encoding and consolidation of declarative memory and spatial memory. The effect of exercise on hippocampal structure and function may be manifested in the proliferation of hippocampal neurons in the brain, the lengthening and increasing of the dendritic spine and then the volume of the hippocampus. According to relevant research reports, somatosensory play significantly increased the volume of CA1, CA4/dentate gyrus (DG), and sub-muscles in the left Hema subregion in a group-dependent manner [44]. In similar studies, the thickness of the bilateral parahippocampal cortex (PHC), the somatosensory cortex (S1), the superior parietal lobe (SPL) and the right insula increased significantly after shooting somatosensory game intervention. The research findings emphasise the specific impact of core game elements such as spatial navigation and visual motor coordination on the structural characteristics of the brain [45]. The study found that the hippocampus or the functional connection of the entorhinal cortex showed growth. At the same time, the benefits of the hippocampus system depended on the navigation strategies used by individuals and the types of games [46]. In addition, many training studies have shown that there is a correlation between the increase of hippocampal volume, the improvement of cognitive function, and the higher level of BDNF after exercise, BDNF activates multiple signaling cascades by binding to the tropomyosin kinase B receptor, leading to regulatory mechanisms for cell proliferation, differentiation, and dendritic branching in the hippocampal subgranular layer. During the 6-week somatosensory play intervention, it was found that the level of BDNF in PD patients increased and participated in the neurogenesis and protection of the hippocampus. At the same time, the gene expression of BDNF in the hippocampus was regulated to increase the level of circulating BDNF [44]. In conclusion, somatosensory play induces the increase of hippocampal volume, which is an effective way of cognitive neuron plasticity. At the same time, BDNF is an important mediator to improve hippocampus plasticity.

##### Prefrontal Cortex

The prefrontal cortex (PFC) is responsible for advanced motor control, planning, and executive behaviour, and is one of the important brain regions involved in memory. It plays an important role in working, episodic and implicit memory. Its anatomical structure includes the following main parts: dorsal prefrontal lobe, anterior cingulate gyrus, medial frontal lobe and orbital cortex [47]. The volume of the prefrontal cortex, like the hippocampus, gradually decreases with age, thereby affecting the cognitive performance of the elderly. Patients with prefrontal cortex damage exhibit abnormalities in emotional and social aspects. Research has found that three-month exercise intervention leads to an increase in the volume or thickness of gray matter in the prefrontal cortex, suggesting that short-term exercise intervention can induce prefrontal plasticity related to cognitive ability in older adults [48]. There is a positive correlation between the baseline aerobic adaptability of older adults during aerobic exercise and the larger thickness of the dorsolateral prefrontal cortex [47]. Involving situational memory, processing speed and execution functions. As is well known, physical exercise can reduce stress and anxiety in older adults. Meanwhile, the prefrontal cortex is involved in processing emotions and social experiences. Long term yoga practice may lead to the best regulation of the emotion and mood of the practitioner, which may cause effective activation of the dorsolateral prefrontal cortex, thereby affecting executive function, especially working memory [49]. Aerobic exercise can reduce the regional homogeneity of the prefrontal cortex, increase its functional specialisation and intrinsic activity intensity and improve cognitive function. Resistance movement has a positive functional change in the hemodynamic activity of the cortical areas related to response inhibition processing: the left anterior insula extends to the lateral orbitofrontal cortex and the front of the left middle temporal gyrus, which indicates that the ability of the elderly to respond to inhibition process is enhanced [50]. The involvement of exergames in the bilateral reduction of prefrontal brain activity and increased asymmetry of hemispheric prefrontal activity reduces oxygenation in the left and right hemispheres. This can reduce the demand for prefrontal resources related to executive function and attention, thereby enabling older adults to benefit from cognitive resources [51]. In summary, somatosensory gaming may improve the plasticity of the prefrontal cortex, thereby affecting cognitive function in older adults.

##### Basal ganglia

The basal ganglia is a general term for the five subcortical nuclei, including the caudate nucleus, putamen, globus pallidus, hypothalamic nucleus, and substantia nigra. The basal ganglia are involved in motor control and learning and the circuit goes from the cortical area to the basal ganglia and back to the cortex. As the structure and function of the motor cortex, cerebellum and basal ganglia pathways decline, and the availability of neurotransmitters decreases, the elderly increasingly rely on cognitive brain processes for motor control. Basal ganglia disorders are Parkinson disease and Huntington's chorea [42,52]. Research reports that the basal ganglia play a central role in regulating the ability to respond and choose, which is crucial to mental flexibility. At the same time, the level of physical fitness also has a positive impact on the conversion of cognitive performance tasks [42]. At the same time, the volume of the caudate nucleus, putamen and globus pallidus was positively correlated with the accuracy of task switching. The volume of the caudate nucleus was a significant mediator between cardiorespiratory health and task-switching performance [53]. However, there is currently very little research on the relationship between cognitive function and basal ganglia in older adults. Although exergames may improve physical fitness in older adults, this is only a mediating pathway, and more research is needed to explore the benefits of exergames directly improving basal ganglia.

**Figure 2:**
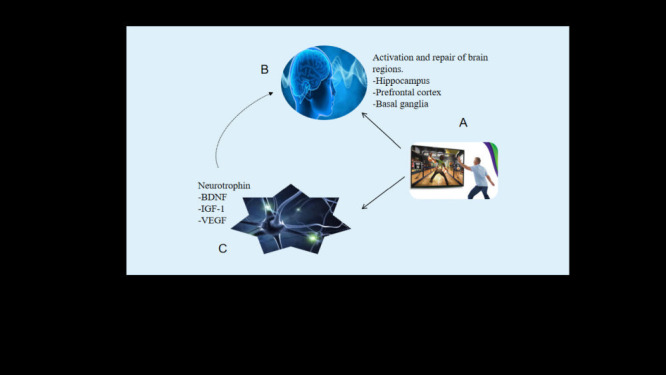
Possible mechanism of exergames improving cognitive function in elder adults.

In conclusion, exergames play affects the cognitive function of the elderly by improving the structure of the hippocampus, prefrontal cortex and basal ganglia, plus the protective effect of neurotrophin. The path is shown in **Figure 2**. In addition, the stimulation effects of different types and dosages of exergames should vary, although their effects on the brain structure of older adults may also vary. There is limited research data on brain structures such as basal ganglia, and optimising exercise prescriptions can enhance research in this area in the future.

### Empirical study on improving cognitive function of older adults through exergames

Executive function (EF) is the highest control center for cognitive function, mainly controlled by the prefrontal lobe, and has functions such as interpretation, perception, inhibition, decision-making and memory retrieval. Cognitive components mainly include working memory and inhibition control [54]. When executive function is impaired, individuals may have difficulty concentrating on a certain point, may experience behavioural reactions that deviate from the target and symptoms, such as sustained memory decline [55]. If there is no timely intervention and prevention, it will lead to the inability of thinking and behaviour to coordinate and adapt to changes in the environment, and gradually develop into diseases such as Alzheimer disease (AD), which has a significant impact on the quality of life of middle-aged and older adults.

#### Effects of somatosensory play on the working memory of the elderly

Working memory refers to the process of maintaining and transforming online information, which is one of the core components of cognitive function. It is related to many high-level cognitive abilities and plays a very important role in learning and daily life. However, with the aging process, its ability also decreases [56]. Working memory is divided into a speech loop, visuospatial template, event buffer and central processing system. Compared with short-term memory which only stores information, it is more complex, because working memory cannot only store but also process information. According to research reports, the elderly's working memory task performance has improved 23 hours later after receiving the intervention of racing exergames [57]. 3D video games have improved the multi-task level of the elderly, expanded the training benefits to the field of working memory and can maintain the training benefits for a while after stopping training [58]. Wang Liyan et al. [59] found that after exergames were involved in older adults, their brain activity patterns were synchronised by low-frequency alpha events in the parietooccipital lobe and frontal lobe. However, the increase of high-frequency alpha desynchronisation was completed in the left hemisphere, which suggested that somatosensory play could improve the working memory of the elderly. As one of the components of executive function, working memory, 16 weeks of physical games such as taijiquan and dance have improved the inhibition control and working memory of the elderly [13]. Somatosensory play may be a better choice to improve working memory. In the comparative study, somatosensory play is more effective than aerobic dance training in improving the working memory of the elderly [60]. Exergames may improve cognitive performance by improving neural work efficiency, which may be better than traditional physical exercise [61]. Eight somatosensory game intervention courses found that the improvement of working memory also seemed to have a positive impact on the elderly's mood [62]. Similar studies have also found that by increasing their attention and memory levels, their levels of depression, anxiety and apathy are reduced [63]. Therefore, exergames not only improve cognitive function but also have a positive effect on their daily life abilities. Compared to other memory abilities, exergames can also play a positive role. Research shows that 6-week Xbox 360 Kinect somatosensory gaming increases BDNF concentration, thereby improving delayed recall ability in older adults [21]. Exergames have a certain interesting nature. Stroke patients not only enjoy themselves but also improve their memory ability by increasing brain activity and increasing blood flow to the affected area [64]. In conclusion, the effect of exergames on working memory is not only positive but also likely to improve the memory function of the elderly from multiple benefits. These studies on the improvement of working memory in the elderly by exergames are shown in **Table 1**.

**Table 1:** Related research of exergames on working memory of the elderly

Author/country	Sample size/age	Intervention time/frequency	Evaluation method	Type of somatosensory game	Findings
Anguera JA et al., America	46/60	60 minutes per session, 3 times per week	EEG testing	Racing Games	Enhanced midline frontal lobe θ Power and Afterload θ relevance.
Wang Li Yan et al., China	30/60	30 minutes per session, 3 times per week	EEG testing	Dance	Synchronisation of low-frequency alpha and high-frequency alpha.
Adcock M et al., Switzerland	40/60	40 minutes per session, 3 times per week	WMS-R	Dance, Tai Chi	The volume of gray matter in frontal lobe and hippocampus was significantly reduced, and working memory was effectively improved (*P* = 0.015).
Zhao, C et al., China	55/60	75 minutes per session, 3 times per week	N-back	Nintendo Wii	Long term exercise training can improve the elderly's working memory (accuracy of 1-back test: ES = 0.76, *P* < 0.01).
Liao YY et al., China	25/65	60 minutes per session, 3 times per week	N-back	Kinect	Working memory performance improved (n-back task test score improved).
Moret B et al., Italy	38/65	45 minutes per session, 3 times per week	N-back	Xbox-360 Kinect	Positive trends were observed in N-back tasks, and working memory improved.
Jahouh M et al., Spain	40/75	45 minutes per session, 3 times per week	MCE	Nintendo Wii	Nintendo Wii improved cognitive performance and depression in elderly people.
Monteblanco et al., Brazil	9/71	40 minutes per session, 2 times per week	MoCA	Xbox-360 Kinect	Promote the enhancement of BDNF and enhance cognitive abilities in elderly individuals in an acute and delayed manner.
Chenchang Xiang, China	6/55	30 minutes per session, 3 times per week	RBMT-II	Ski Sensation Games	Exergames interactive games can improve memory function in stroke patients.

WMS-R – the Wechsler Memory Scale-Revised, n-bac – the spatial n-back task test, MoCA – Montreal Cognitive Assessment, RBMT-II – rivermead behavioural memory test second edition

#### The impact of exergames on inhibitory control in older adults

Due to the need for decision-making, mental flexibility, planning and inhibitory control in games, the executive function is the main cognitive function of the somatosensory game simulation. Numerous studies have confirmed that sensory gaming is an effective measure to improve executive function [47,61]. Effective improvement in inhibition control in older adults [60,62]. Family based physical games such as Tai Chi and dance intervene in older Adult [13]. The duration of each intervention was 30-40 minutes, lasting for 16 weeks, and it was found that somatosensory gaming significantly improved the inhibitory control level of the elderly. At the same time, it effectively reduces the risk of falls in daily life, thereby improving the daily performance of the elderly. After receiving Nintendo Wii training, the executive function of older Adult has improved, with a moderate to the high magnitude of effect, and has reduced adverse sedentary behaviour in older adults [65]. The regulation of prefrontal oxygenation induced by exergames is related to the improvement of executive function. Elderly participants received 8-week dance-like exergames and found that during rapid movement, bilateral prefrontal brain activity decreased, while hemispheric prefrontal cortex activity increased asymmetrically [51]. Tai Chi, as one of the effective measures to improve cognitive function in older Adult, involves using Kinect to participate in dual-task interactive Tai Chi. It has been found that Tai Chi in exergames effectively improves executive and physical functions in older adults [66].

The impact of exergames on the executive function of patients with special diseases is also positive and exergames are one of the clinical rehabilitation interventions for stroke patients. Exercise lower limb muscle strength and balance ability through changes in body posture. Entertainment projects can stimulate the brain of older adults, increasing their brain blood flow. Elderly people participating in exergames with their families can actively alleviate negative emotions such as depression and anxiety, thereby improving their executive function [67]. Therefore, a comprehensive rehabilitation that integrates emotions, physical function and social support will be more conducive to improving the executive function level of the elderly. A study found that cognitive ability is closely related to posture control systems and higher cognitive ability may effectively prevent falls [68]. Although the frequency of postural swaying is associated with lower cognitive states, it has been found through Nintendo Wii Balance Board (WBB) intervention in elderly individuals at risk of falls that improved cognitive abilities can effectively prevent falls [69]. In addition, the volume and thickness of the prefrontal lobe are related to executive function and the smaller volume of the prefrontal region may lead to slower gait speed through slower information processing [70]. From this, it can be seen that exergames improve gait and balance by executing functions, and prevent falls in older adults, which is the key to healthy aging. These studies on the improvement of inhibitory control in the elderly by exergames are shown in **Table 2**.

**Table 2:** Research on the inhibitory control of exergames in elderly people

Author/country	Sample size/age	Intervention time/frequency	Evaluation method	Type of somatosensory game	Findings
Moret B et al., Italy	38/65	45 minutes per session, 3 times per week	Stroop-CW	Xbox-360 Kinect	The suppression control has been improved.
Liao YY et al., China	25/65 _~_ 90	60 minutes per session, 3 times per week	EXIT-25	Kinect	Through 12 weeks of exercise games, the overall cognitive and executive functions of frail elderly people are enhanced.
Zhao, C et al., China	55/60	75 minutes per session, 3 times per week	Stroop Test	Nintendo Wii	Exergames can effectively improve the inhibitory control of executive function in elderly people.
Adcock M et al., Switzerland	40/60	40 minutes per session, 3 times per week	VST	Dance, Tai Chi	The volume of gray matter in frontal lobe and hippocampus was significantly reduced, and executive function was significantly improved.
María Carrasco et al., Spain	22/65	60 minutes per session, 2 times per week	TMT	Nintendo Wii	Positive differences can be observed in the B section of TMT, indicating an improvement in executive function.
Eggenberger P et al., Switzerland	33/65	30 minutes per session, 3 times per week	MoCA	Positive Gaming	Sensory gaming enhances the adaptation of the prefrontal lobe, thereby improving inhibitory control.
Kayama H et al., Japan	41/65	75 minutes per session, once a week	VFT	Tai Chi Kinect	Improved executive function and leg muscle strength in the elderly, effectively preventing falls.
Leach JM et al., Italy	30/65	Ominous	CFL	Nintendo Wii balance board	Posture control is related to cognitive ability, and Nintendo Wii has improved posture control ability.
Schättin A, Italy	79/65	30 minutes per session, 2 times per week	TAP	Dance	Exergames have a positive impact on the activity and/or function of the prefrontal cortex.

VST – the Victoria Stroop Test, EXIT-25 – the executive interview 25, Stroop-CW – stroop color-word, TMT – the trail making test, MoCA – Montreal Cognitive Assessment, VFT – verbal fluency test, CFL – letter fluency, TAP – the test battery test for attentional performance

In summary, exergames games stimulate the cognitive function and overall muscle activity of the elderly through different sound, light and screen interactions. In addition, the victory or defeat of exergames is also the key to the self-confidence and fun gained by the elderly in the process of participating in the interaction. Many colorful spare time lives, combined with increased physical activity, can activate the brain and maintain cognitive function, which is reflected in older Adult's working memory and inhibition control.

## DISCUSSION

This article only focuses on the impact of exergames on executive function in elderly cognitive function, without exploring the impact of other cognitive components, and does not discuss the impact of different types of exergames and exercise dose effects on elderly cognitive function, which is one of the limitations of this article. Therefore, it is suggested that in the future, different types of exergames and exercise intensity can be utilised to explore the impact of body cognitive training combined with personalised optimal training on different cognitive levels and body functions of older adults, as well as measurable changes in brain structure. In addition, disease populations can be further categorised, such as stroke patients and high-risk fall patients, to study the cognitive performance improvement of exergames on special patients. Especially in terms of neuronal outcomes, exercise games with different metabolic and cognitive needs may trigger different mechanisms. It is worth noting that when implementing exergames, it is necessary to scientifically evaluate the training conditions and requirements of the elderly, respect their physiological differences (decrease in visual ability and physical function) and design exercise prescriptions for exergames based on different mental and operational abilities, to achieve the maximum optimisation benefits of the elderly's health status.

## CONCLUSIONS

This article reviews the impact of elderly participation in exergames on cognitive function through a literature review. It was found that somatosensory play improved the elderly's working memory and executive function and supported the relationship between somatosensory play and the elderly's cognitive performance. At the same time, exergames are also an effective means to improve the health status and maintain life independence of the elderly. From the perspective of mechanism, somatosensory play promotes the signal transduction of brain neurotrophin, thereby protecting the structure and function of neurons in specific brain regions, and delaying the decline of cognitive function of the elderly as far as possible under aging and neuropathological conditions. Therefore, it is recommended to use exergames as an effective means for the physical and mental health development of the elderly.
